# High-Throughput Customization of Plant Microbiomes for Sustainable Agriculture

**DOI:** 10.3389/fpls.2020.569742

**Published:** 2020-09-08

**Authors:** Jianfeng Du, Yang Li, Ziyi Yin, Hongfeng Wang, Xiaoying Zhang, Xinhua Ding

**Affiliations:** ^1^ State Key Laboratory of Crop Biology, Shandong Provincial Key Laboratory for Biology of Vegetable Diseases and Insect Pests, College of Plant Protection, Shandong Agricultural University, Tai’an, China; ^2^ Shandong Pengbo Biotechnology Co., Ltd., Tai’an, China

**Keywords:** highthroughput, plant pathogen, plant–microorganism relationship, synthetic microbial community, sustainable agriculture

## Abstract

Soil microorganisms can form a stable dynamic system with plant root systems. The composition of the soil microorganism community is related to the growth and stress resistance of plants; in turn, soil microorganisms are also regulated by plant genotypes and root exudates. Therefore, research on how to identify microorganisms that are beneficial or harmful to plants, study the interaction between microorganisms and plants, and form stable microbial communities for better plant growth plays an important role in sustainable agriculture. It is of great significance to identify and analyze rhizosphere microorganisms and plant endophytes through high-throughput methods, especially to analyze which microorganisms are beneficial to plants, which are harmful to plants, and which are opportunistic pathogens. This review provides a theoretical basis and outlook for the utilization of beneficial microbes in sustainable agriculture.

## Introduction

Soil is an important medium in which plants survive and grow. A network of hormonal and other responses with respect to soil conditions is involved in attuning the growth and development of a plant to its environment (Passioura, 2002). The composition of microorganisms in the soil is closely related to plant health (Chaparro et al., 2012; Yang et al., 2019). For example, rhizosphere microorganisms can not only promote plant growth but also improve the drought resistance of crops (de Vries et al., 2020). Coevolving interactions between a plant population and its microbiota can potentially yield a rhizosphere enriched in metagenomes containing blueprints for a vast array of natural products (Carver et al., 2016).

In recent years, the balance of microbial communities in the soil has been disrupted due to large-scale use of fertilizers, pesticides, single planting models, and fire burnings, which seriously threaten agricultural production, human health, and global ecological security. Microorganisms, which vary tremendously in diversity and quantity, are an indispensable part of the soil ecosystem and are involved in several processes, including material circulation, energy conversion, and plant health (Shen, 2008; Zhang et al., 2019). For example, green tuff fertilizer application influences soil microorganisms, plant growth, and soil chemical parameters in green onion (*Allium fistulosum L*.) cultivation (Kuroda et al., 2020). The microorganisms in the soil consist of both beneficial and harmful microorganisms (Cheng et al., 2018). Two of the key functions of soil microorganisms are to promote plant health and increase soil productivity. Some indigenous microorganisms of contaminated soil systems also have the capability of degrading soil contaminants and thus are frequently used for bioremediation purposes (Dubey et al., 2020). The metabolism of beneficial microorganisms can provide a source of materials for plants, including nitrogen, phosphorus, potassium and other elements, to improve soil fertility and promote plant growth and health (Lu et al., 2019). For example, soil microorganisms can convert inert atmospheric nitrogen into ionic nitrogen, which is directly absorbed by plants through a series of chemical processes and ultimately provides more sufficient nutrients for plant growth than other forms (Jahan et al., 2016). The importance of biological nitrogen fixation as a sustainable nitrogen source for plants has also been proven (Zilli et al., 2019). Moreover, beneficial soil microorganisms can improve the soil microenvironment, promote plant growth, and reduce the risk of infection by pathogenic bacteria. *Pseudomonas fluorescens* 1-8 has a strong preventive effect against *Fusarium* root rot (Wang et al., 2015). Extracts of the endophyte *Paecilomyces variotii* (ZNC) can promote plant growth and improve plant resistance to several kinds of pathogenic bacteria and viruses (Lu et al., 2019; Peng et al., 2020). The combined effects of environmentally detrimental changes in local land use and alterations of the global climate disrupt natural ecosystems and can increase the risk of disease transmission (Patz et al., 2000). Significant microbial community shifts due to no tillage, cover crops, and N fertilization (Mbuthia et al., 2015). Daayf et al. (2003) revealed that *Bacillus*, *Pseudomonas*, *Rahnella*, and *Serratia* can control late blight of potato caused by *Phytophthora infestans* (strain US-8) through antibiosis or the induction of plant defense systems (Daayf et al., 2003). Furthermore, beneficial microorganisms can also decompose insoluble minerals in the soil and convert them into soluble mineral compounds. *Bacillus circularis* Z1-3 has the ability to dissolve potassium (Lynn et al., 2013). *Pantoea* J49 functions in dissolving phosphorus and can increase the biomass of peanut plants (Taurian et al., 2009). Several soil-borne microbes, such as mycorrhizal fungi and plant growth-promoting rhizobacteria, can help plants deal with biotic and abiotic stresses *via* plant growth promotion and induced resistance (Pineda et al., 2010). Beneficial microorganisms can secrete plant growth hormones, which can not only effectively promote the growth of plants but also effectively improve the ability of plant roots to absorb various trace elements from soil (Ma et al., 2018b). Indole-3-acetic acid (IAA)-producing bacteria (*Pseudomonas* sp. and *Burkholderia* sp.) can promote the root growth of *Arabidopsis* seedlings in natural soil (Jiang et al., 2012). Soil bacteria transform atmospheric N_2_ into ammonia and are central to soil and plant health. They play a pivotal role in the cycling of nutrients within the soil (Hayat et al., 2010). However, harmful microorganisms can reduce plant growth and the yield and quality of crops and even leave toxins in crops, endangering human health (Song et al., 2016). *Fusarium oxysporum* f. sp. *radicis-lycopersici* can cause *Fusarium* crown and root rot in tomato cultivation (Szczechura et al., 2013). *Fusarium* head blight of wheat can reduce the yield of wheat by 10–20% and even 80–90% in severe cases and can lead to crop failure in severely diseased fields. Moreover, the vomitoxin produced by this pathogen seriously adulterates food (Dweba et al., 2017). Bacterial wilt, caused by *Pseudomonas solanacearum*, can harm many crops, such as tomato, eggplant, pepper, and potato (Hayward, 1991).

In environments conducive to healthy plant growth, there are certain proportions of beneficial and harmful microbes in the microbial community (Xia et al., 2016). Once these proportions are disrupted, plants will become diseased. Studies have shown that several genes in plants can be modified to regulate the steady state of the microbiome or optimize the microbiome to improve plant health and resistance to stressful environments, thereby increasing the yield of important crops and improving natural ecosystems (Chen T. et al., 2020). Simultaneously, the composition and stability of the microbial community are also closely related to the healthy growth of plants (Xia et al., 2016). Therefore, studying the microbial community is very important for determining whether certain microorganisms are beneficial or harmful to plants, how to synthesize a stable microbial community for plants and how to study the interaction between microorganisms and plants. The plant microbiota can fundamentally change agriculture and ensure agricultural development in a more precise direction. In addition to addressing the current challenges in crop production, we urgently need to apply new microbial agents to agricultural production. In this paper, we hope to provide a theoretical basis for sustainable agriculture through high-throughput customization of the plant microbiome as well as to identify pathogens and study the relationship between microorganisms and plants. This review provides a theoretical basis for the use of a customized plant microbiome in sustainable agriculture.

## The Development of Traditional Agricultural Microbial Preparations

Beneficial microorganisms are essential for agricultural production and can promote plant growth and protect plants. For example, *Bacillus subtilis* SYST2 can promote tomato growth (Tahir et al., 2017). Microbial populations are instrumental to fundamental processes that drive the stability and productivity of agroecosystems. Several investigations have aimed to improve the understanding of the diversity, dynamics and importance of soil microbial communities and their beneficial and cooperative roles in agricultural productivity. However, Singh et al. (2011) described only the contributions of plant growth-promoting rhizobacteria (PGPR) and cyanobacteria in safe and sustainable agricultural development. Therefore, it is very important to screen beneficial microorganisms and reveal their mechanisms of action for the development of modern agriculture. At present, traditional microbiological techniques, such as antagonism tests, are used to test microbial functions. For example, *Bacillus velezensis* had strong inhibitory effects against *Ralstonia solanacearum* and *Fusarium oxysporum* (Cao et al., 2018). Husen (2016) used gas chromatography to test the ability of bacteria to fix nitrogen (Husen, 2016). Many rhizospheric bacterial strains possess plant growth-promoting mechanisms. These bacteria can be applied as biofertilizers in agriculture and forestry, enhancing crop yields. Bacterial biofertilizers can improve plant growth through several mechanisms: (i) the synthesis of plant nutrients or phytohormones, which can be absorbed by plants; (ii) the mobilization of soil compounds, making them available for plant use as nutrients; (iii) the protection of plants under stressful conditions, thereby counteracting the negative impacts of stress; or (iv) defense against plant pathogens, reducing plant diseases or death (García-Fraile et al., 2015). Moreover, the verification of beneficial microbial functions is mostly performed using one or a few phenotypes, such as biomass, and the control of disease effects. For instance, Zhao et al. (2018) screened functional strains based on the juvenile mortality of *Meloidogyne incognita* and on the number of these nematodes in soil (Zhao et al., 2018). However, the effect of beneficial microorganisms on the microbial community and the stability of the newly formed microbial community have been somewhat ignored in the process of screening functional microbes. The mechanisms by which microorganisms benefit plants are mostly studied in terms of metabolites or functional genes. It was proven that *Bacillus aryabhattai* SRB02 can promote plant growth by producing abscisic acid, IAA, cytokinin and gibberellin (Park et al., 2017). Additionally, *Bacillus flexus* KLBMP 4941 can improve the growth of host seedlings under salt stress. Through genome-wide analysis, genes for high salt tolerance were also found in the KLBMP 4941 genome (Wang et al., 2017). When microbiological preparations are developed *via* traditional microbiological techniques, only beneficial microorganisms are screened, and fermentation technology is used. However, these traditional microbiological techniques for developing microbial preparations do not consider whether beneficial microorganisms can perform their functions under different selection pressures and compare with those functions with those of the original microorganisms. Moreover, these techniques do not focus on the functional principles of corresponding strains from the overall perspective of plant and microbial communities in screening and verifying beneficial microorganisms, leaving the relationships between functional strains, microbial communities and plants unclear. Considering the challenges in working with traditional microbial inoculants, including instability in field trials, lack of uniformity in inoculum composition, and weak rhizosphere competitiveness, it seems important to solve the above problems using synthetic microbial communities. Regardless of whether the community can form a stable relationship with microorganisms in soil and with plant, the time and money spent on these issues are far greater than the cost of using synthetic microbial communities to develop microbial preparations. The importance of microorganisms for the plant growth and functions will be discussed in the following section.

## The Plant Microbiome

Healthy plants harbor complex microbial communities that play an important role in plant growth and health. These microbial communities have been selected by plants over a long period of time to form a plant-specific microbiome (microbiota), which mainly depends on the diversity of microbes widely present in natural soil (Bai et al., 2015). Recently, several researchers have focused on the plant microbiome and reported enlightening results. For example, Beattie (2015) isolated and cultivated up to 65% of the bacteria in the root system and up to 54% of the bacteria in the leaf microbial community of *Arabidopsis thaliana* grown in nature and established a resource library of these strains to reconstruct leaf and root microorganisms in sterile plant groups. The research showed that the compositions of the bacterial communities produced in the laboratory and in nature have high similarity, which created opportunities for the field of microbiome reconstruction. Such a defined microbiome allows us to control the disturbance of the microbiome for the first time under controlled environmental conditions compared to the unpredictable changes that inevitably occur in nature due to complex environmental fluctuations (Beattie, 2015). At the same time, many researchers have found that the microbiome is closely related to plant immunity and phenotypic characteristics such as resistance and yield. For example, through drought-induced enrichment and exchange conversion analysis of metabolites between plants and microorganisms, it was revealed that the plant microbiome could improve plant adaptability, and crop yields could be increased significantly through this approach (Xu et al., 2018). It was also shown that protists are an important part of the rhizosphere microbiome, and the interaction between predators and prey greatly improves our prediction and determination of the microbiome’s roles in plant health (Gao et al., 2019). Beneficial microorganisms can improve crop health in environments conducive to the growth of beneficial microorganisms, and plants’ unique microorganisms can promote their survival and yield in harsh environments. For example, the plant microbiome not only improves crop yield but also enhances resistance to different stresses, such as drought (Zhao et al., 2017). Consistent with this, weeds can be controlled by regulating the plant microbiome, and this strategy not only limits economic costs but also protects the environment (Masteling et al., 2019). Therefore, the plant microbiota can fundamentally change agriculture and ensure its development in a more precise direction. Meanwhile, studies on the relationships between microbial communities and plants can not only allow the precise regulation of microbial communities but also play an essential role in the sustainable development of precision agriculture. Therefore, in the following sections, we will discuss how the high-throughput methods can be used to identify beneficial plant microbiome and to study plant-microbiome interactions.

## Identification of Microorganisms by High-Throughput Methods

Both harmful and beneficial bacteria and fungi need to be considered, especially with regard to how they communicate with plant hosts, such as providing biological nutrient sources or suppressing the host’s immune response (Zhu et al., 2018). Endophytes are similar to pathogens, having the ability to enter the host tissue and stay there without causing harm to the host and even being beneficial (Ma et al., 2018a). Both partner plants and microorganisms, such as fungi, benefit from the relationship: mycorrhizal fungi improve the nutrient status of their host plants, influencing mineral nutrition, water absorption, growth and disease resistance, whereas in exchange, the host plant is necessary for fungal growth and reproduction (Bonfante and Genre, 2010). Therefore, beneficial and pathogenic microorganisms exhibit common physiological characteristics and evolutionary similarities (Gong et al., 2016). To a certain extent, the performance of pathogenic phenotypes may depend on small differences in microorganisms and sometimes even on the host. Microbial secretions and the number and nature of secretory effectors may constitute important distinctions between beneficial bacteria and pathogens (Rodriguez et al., 2019). High-throughput technology that can identify thousands of taxa in each of hundreds of samples at the same time is convenient for studying any organism and pathogenic group at the same time, greatly expanding our knowledge of plant diseases (Yan et al., 2018). Currently, the high-throughput technologies most commonly used to identify diseases are real-time quantitative PCR (RT-qRCR), droplet digital PCR (ddPCR), gene chips, macro barcodes, metagenomics, and metatranscriptomics (Gong et al., 2016). While RT-qPCR and ddPCR can detect the number of microorganisms, they detect only specific microorganisms. These techniques are also used to determine the relative and absolute abundances of pathogenic microorganisms (Schena et al., 2004; Hindson et al., 2011). For example, Gehesquière et al. (2013) used RT-qPCR to detect *Cylindrocladium buxicola* in plants (Gehesquière et al., 2013). Research has shown that the accuracy of ddPCR is much higher than that of ordinary RT-qPCR (Gutiérrez-Aguirre et al., 2015). ddPCR showed significantly improved analytical sensitivity compared to that of RT-qPCR and improved the detection of low concentrations of *Ralstonia solanacearum* in potato tuber samples (Dreo et al., 2014). The shortcomings of using RT-qPCR and ddPCR, such as the ability to detect only specific microorganisms, are overcome by the use of more advanced molecular tools, such as gene chips, macro barcodes, metagenomics, and metatranscriptomics. The monitoring of the presence and abundance of specific pathogenic groups and pathogenicity-related genes is mainly achieved through gene chips and high-throughput technologies. Mendes et al. (2011) identified rhizosphere microbial taxa and specific genes with antagonistic effects on the fungal root rot pathogen *Rhizoctonia solani* (Mendes et al., 2011). Since gene chips have shortcomings, including a lack of detection of most target species and their functions in specific target environments and various suboptimally specific probes, high-throughput technologies are currently used. Macro barcodes, metagenomics, and metatranscriptomics can be used to analyze most species and related genes, and metagenomics can be used to effectively analyze the expression of related resistance genes in most species (Tedersoo et al., 2019). High-throughput metagenomics has been used to detect the relative abundance of soil pathogens in grasslands (Cline et al., 2018). It is also possible to predict plant pathogens with the help of databases such as FUNGuild and INSDc (Nguyen et al., 2016). Metagenomics and metatranscriptomics can also be used to detect unidentified pathogens. Therefore, high-throughput techniques can provide powerful tools for determining plant health.

## Using High-Throughput Methods to Study the Relationships Between Plants and Microorganisms

Beneficial microorganisms resist harmful microorganisms in the rhizosphere in various ways and promote plant colonization, growth and development. Plants mainly affect the microbial community structure of the rhizosphere through rhizosphere secretions and improve the nutrient utilization rate to enhance resistance to disease and stress. Members of the microbial community can also promote plant growth and health through their own metabolic activities. Microorganisms play a key role in the functioning of plants through their positive impacts on growth and development. In general, rhizosphere microorganisms promote plant growth directly by providing plants with minerals such as nitrogen and phosphorus and by synthesizing growth regulators, as well as indirectly by inhibiting the development of various plant pathogens (Woźniak and Gałązka, 2019). Plants rely to a large extent on their root microbiota to absorb nutrients and resist stress. Plants subjected to biotic or abiotic stress elicit a “cry for help” by adjusting their root exudate composition. Differential root exudates may be able to regulate the rhizosphere microbiome, promote the members of the microbiota to help plants absorb nutrients and water, or protect plants from invasive species. Therefore, we can use high-throughput methods, such as metagenomics, 16S rRNA gene sequencing, 18S rDNA/ITS gene sequencing, and genome-wide expression analysis, to more thoroughly understand the relationships between microbial communities and plants from the synthetic microbial community and to explore the “cry-for-help” hypothesis. This hypothesis provides a reasonable mechanism for the feedback response of soil to plant diseases (Rolfe et al., 2019), motivating studies on the relationships between microorganisms and root exudates *via* metabolomics and high-throughput analysis. According to 16S rRNA sequencing and gas chromatography-mass spectrometry (GC-MS) analysis, plants have been shown to recruit probiotics through root exudates to promote their own growth and disease resistance (Yuan et al., 2018). Chen Y. et al. (2020) used metabolomics and high-throughput analysis to prove that cyanide changed the microbial composition of peanuts and reorganized the rhizosphere microbial symbiotic network of peanuts by changing the abundance of actinomycetes. The reorganized rhizosphere microbiome provided more effective nutrients for the peanut root system (Chen Y. et al., 2020). Through high-throughput sequencing and metabolomics, it was confirmed that 6-methoxy-benzoxazolin-2-one indirectly changed the root-associated flora (Hu et al., 2018). The relationships between different strains and metabolites can also be explored by mathematical models such as the MelonnPan model. For example, how the root system recruits beneficial flora can be studied by specialized devices, such as using the olfactory system to ingeniously evaluate the ability of root volatile organic compounds (VOCs) to recruit soil microorganisms (Schulz et al., 2018).

Synthetic microbial communities are powerful tools for studying the relationships between microbial communities and plant phenotypes. The causal relationships between the members of the microbial community and the host phenotype were successfully inferred with a small synthetic community designed by plant-bacteria binary association analysis (Herrera Paredes et al., 2018). Using the root-associated microbial communities of three *Arabidopsis* populations, Durán et al. (2018) found that most of the bacteria and filamentous microorganisms were negatively correlated. Through recombination experiments, they found that the bacterial microbiome was essential for the survival of the plant and the protection of root-associated filamentous eukaryotes (Durán et al., 2018). The connection between nutrition and defense has been proven by combining 16S rRNA gene sequencing, genome-wide expression analysis, synthetic community (SynComs) analysis and modeling, as well as functional analysis (Castrillo et al., 2017), suggesting that the relationships between microbial communities and plants can be better understood by employing multiple omics approaches. Therefore, studying the relationships between microbial communities and plants can aid in the precise regulation and customization of an exclusive plant microbiome and can provide useful information for future agricultural developments, which will be elaborated in the following lines.

## Customization of the Plant Microbiome With High-Throughput Methods

Synthetic microbial communities (SMCs) are a new microbial-community-level application of synthetic biology. SMCs can shed light on interspecies interactions and microbial regulatory mechanisms as well as perform specific functions. Furthermore, SMCs have low complexity, high controllability, good stability, and other advantages. In recent years, high-throughput technology has provided powerful tools for customizing exclusive plant microbiomes. Two main methods are currently used to customize the microbiomes of plants: top-down SMCs and bottom-up SMCs (Liang et al., 2019). The application of top-down SMCs involves using external stimuli to regulate the plant microbial community and analyzing the structure and function of the microbial community by high-throughput methods such as metagenomics, 16S RNA sequencing, and 18S rDNA/ITS sequencing. These tools are useful for analyzing the composition of microbial communities in plants with different phenotypes, as well as the assembly mechanisms of microbial communities under different selection pressures, and the findings can be used in combination with microbial isolation and culture technology to synthesize microbial communities.

Studies have shown that external biological stimulation can change microbial community composition, increase the number of beneficial bacteria in soil, reduce plant disease incidence, or induce plant resistance, thus improving crop quality and yield. For example, when *Arabidopsis* is infected by pathogens, it can adjust the microbial community in its rhizosphere and specifically recruit certain beneficial microorganisms with disease resistance and growth promotion effects so that its offspring can survive in the same soil and exhibit enhanced plant resistance (Berendsen et al., 2018). The application of bottom-up SMCs involves using high-throughput sequencing and bioinformatics to predict metabolic networks and the relationships between microorganisms and plants. By combining this technique with strain separation and culture technology, the core microbiome can be constructed according to the main composition, phylogeny, sustainability and coherence of the microbial population. Currently, there are two main strategies for synthesizing microbial communities from the bottom up. Liu et al. (2019) proposed an SMC workflow, but this workflow mainly describes microbial cultivation by high-throughput methods and microbial identification and does not provide a detailed introduction on how to screen and combine microorganisms (Liu et al., 2019). The SMC strategy provided by Qin et al. (2016) is based on the use of high-throughput sequencing analysis to study community structure. By methods such as Venn diagram and network analyses, the core microbiome can be predicted based on the main components, composition, phylogeny, continuity, and coherence of the microbial community. At the same time, by extensive cultivation and inoculation tests, we can identify beneficial strains or flora that determine the phenotype and then combine beneficial microorganisms with different functions and synergistic effects to synthesize the microbial community (Qin et al., 2016). The plant immune system can not only resist plant pathogenic microorganisms but also tolerate beneficial microorganisms. Beneficial microorganisms can coexist with plants. The mechanisms of induced plant immunity can be divided into microbiota-mediated immunity (MMI) and direct microbial competition (DMC). MMI occurs when beneficial microorganisms induce disease resistance in plants. In contrast, DMC occurs when beneficial microorganisms directly develop resistance to plant pathogenic microorganisms. Vannier proposed a strategy for synthesizing microbial communities based on MMI and/or DMC, hoping to promote plant disease resistance and fill the gap in the design of SMCs (SynComs) with plant protective functions (Vannier et al., 2019). The methods provided by Vannier et al. (2019) and Qin et al. (2016) have many similarities, whereas the design–build–test–learn (DSTL)strategy proposed by Lawson is very different (Lawson et al., 2019). The DSTL strategy uses *in situ* transgenic technology and CRISPR-Cas9 to induce precise biofilm formation and manipulate the metabolic network of *in situ* microorganisms, effectively combining self-assembly and synthetic microbiota, and includes top-down and bottom-up design processes, microbial community synthesis and self-assembly construction methods, and emerging tools for analyzing microbiome functions. Top-down SMCs can deepen the understanding of plant immune systems and the mechanisms underlying plant recruitment of beneficial microbes. For example, Illumina MiSeq sequencing of the 16S rRNA gene and the 18S rRNA gene and metagenetic sequencing were used to comprehensively analyze the whole-rhizosphere microbiome, including bacteria, fungi and protozoa, as well as the metabolic genes related to microbial functions (Xiong et al., 2020). Zhu et al. (2020) used metagenomic analysis to identify microbial communities and functional genes related to the degradation of di-(2-ethylhexyl) phthalate in soil (Zhu et al., 2020). The response of microbial communities in tropical soils to the release of phosphorus was revealed by metagenomics, enzyme functional analysis and macrotranscriptomics (Johnston et al., 2019). Macrogenomic guidance analysis and network analysis were used to identify specific groups and functions in order to design microbial groups and obtain specific microbe-driven plant phenotypes (Carrión et al., 2019). Specific bacterial groups with increased salt tolerance were identified, which can be used as biological indicators of high salt tolerance by using multiple mathematical models, such as logistic models, double logistic models and high-throughput models (Rath et al., 2019). A change in resource stoichiometry was found to change the resistance of the community to invasion by having a disproportionate impact on the growth of species (Tianjie et al., 2018). Thus far, we have little understanding of the mechanisms of microbial community action, making it impossible to accurately synthesize microbial communities from top to bottom. For example, metagenomics and network analysis revealed that fungal infections in plant roots are enriched in *Chitinophagaceae* and *Flavobacteriaceae*, as well as *chitinase* genes, nonribosomal peptide synthetases (NRPSs) and various unknown biosynthetic gene clusters of polyketide synthases (PKSs). Strain-specific synthetic chitin-producing and flavobacterial flora can continuously inhibit fungal root disease. Site-directed mutagenesis showed that the previously unidentified NRPS-PKS gene cluster in *Flavobacterium* is essential for disease suppression by the endophyte flora (Carrión et al., 2019). Meanwhile, the rhizosphere microorganisms of a disease-resistant tomato variety (7996) and a susceptible variety (Moneymaker) were different according to 16S and metagenomic sequencing methods. A rhizosphere microbial exchange experiment verified that the soil of disease-resistant varieties can alleviate disease symptoms in susceptible varieties. A new genome of *Flavobacterium* TRG1 was reassembled by analyzing metagenomic data, which revealed that this strain has a greater abundance among disease-resistant varieties than among susceptible varieties. Through verification, it was found that a strain of TRM1 could indeed relieve disease symptoms in susceptible plants (Kwak et al., 2018). Bodenhausen et al. (2014) established a complex synthetic microflora composed of seven strains representing the most abundant phylum in the leaf layer. This microflora was used to study how the composition of the *Arabidopsis* interleaflet microbial community changes with changes in *Arabidopsis* genotype (Bodenhausen et al., 2014). Principal component analysis (PCA) and high-throughput analysis were used to prove that after infection with the downy mildew pathogen, the *Arabidopsis* foliar defense system was activated, and three bacteria were specifically enriched in the rhizosphere. The three beneficial bacteria were subsequently isolated and found to synergistically promote biofilm formation under experimental conditions (Berendsen et al., 2018). Through traditional microbial isolation culture technology, *in situ* culture, and loss cytometry, a large number of microbial resources can be obtained (Berdy et al., 2017; Lagier et al., 2018; Kuete Yimagou et al., 2019; Dance, 2020). The relationships between plant phenotypes and beneficial microbes as well as the assembly mechanisms of the beneficial microbial community under different selection pressures, such as those related to temperature, nutrition, and the number of plant pathogenic microbes, can be understood by applying metagenomics methods and controlled experiments. Second, high-performance liquid chromatography and other technologies can be used to analyze the relationships between plant root secretion metabolites and beneficial microbial communities under different selection pressures and the influence of plant root secretion metabolites on the mechanism driving beneficial microbial community composition (Sasse et al., 2017; Hu et al., 2020; Liu et al., 2020). By introducing beneficial microbes that can perform stable ecological functions into crops under different selection pressures, such as disease-resistant beneficial microorganisms, not only can a number of beneficial microorganisms survive and perform ecological functions in the field but also the root secretion of beneficial microorganisms can more effectively perform ecological functions in the field and ensure the survival of beneficial microorganisms. Although the cost of sequencing is high, other prevention and treatment techniques have many shortcomings. However, direct inoculation with traditional microbial functional preparations may lead to problems such as unstable effects. Although molecular technology is very expensive, sequencing methods provide an understanding of microbial communities. Moreover, traditional culture techniques, *in situ* culture and flow cytometry culture of microorganisms have greatly expanded the scope of our cultivable microorganisms. On the basis of this understanding, combining microorganismal isolation and culture technology to synthesize microbial communities can provide useful insights for modern agriculture. Thus, SMCs are currently being studied and can overcome the problems associated with microbial preparations, such as instability and unsatisfactory effects in field tests. SMCs can elucidate the mechanisms underlying coevolution between plants and root beneficial microbes and provide new ideas for the development of microbial inoculants.

## Concluding Remarks

Although traditional microbial technology has been used to screen many excellent beneficial microorganisms and microbial metabolites for plant growth, it has many limitations, such as not considering the composition, abundance, and status of beneficial microorganisms in the microbial community and the impact of the entire gene expression profile. Moreover, traditional microbial technology has many complex steps, and only a limited number of plant pathogens can be identified when identifying beneficial and harmful microorganisms. In turn, high-throughput methods, such as metagenomics, can greatly overcome the deficiencies of traditional microbial technology. First, high-throughput technology can quantitatively assess community composition and abundance, the entire genome profile, gene expression profiles at the transcript or proteome level, *in situ* metabolites, and spatial information at various scales. Second, high-throughput technologies such as metagenomics can greatly complement traditional microbial technology in the detection of microorganisms and expand these detection capabilities. High-throughput technology can be employed to identify uncultivable microorganisms. Third, synthetic plant microbial communities customized with high-throughput methods can address problems commonly faced with microbial fertilizers, such as the mutability of functional strains and a better understanding of the relationship between microbial communities and plants. CRISPR-Cas9 gene editing technology can also be used to genetically modify the synthetic microbial community in order to study the relationships between different gene clusters and plant phenotype. In short, the use of high-throughput technology to synthesize microbial communities for use in plant production represents a new method of developing microbial preparations. This method can provide excellent microbial strains to support modern agricultural production.

## Author Contributions

XD and JD designed and wrote the manuscript. YL, ZY, HW, and XZ provided suggestions on the writing of the manuscript. All authors contributed to the article and approved the submitted version.

## Funding

This work was supported by the National Natural Science Foundation (31872925), Shandong Province Key Research and Development Plan (2019JZZY020608, 2019GNC106152), Natural Science Outstanding Youth Fund of Shandong Province (JQ201807), and Science and Technology Support Plan for Youth Innovation of Colleges and Universities of Shandong Province (2019KJF023).

## Conflict of Interest

HW and XZ are employed by Shandong Pengbo Biotechnology Co., Ltd.

The remaining authors declare that the research was conducted in the absence of any commercial or financial relationships that could be construed as a potential conflict of interest.
